# Differences in the Cardiometabolic Control in Type 2 Diabetes according to Gender and the Presence of Cardiovascular Disease: Results from the eControl Study

**DOI:** 10.1155/2014/131709

**Published:** 2014-09-21

**Authors:** Josep Franch-Nadal, Manel Mata-Cases, Irene Vinagre, Flor Patitucci, Eduard Hermosilla, Aina Casellas, Bonaventura Bolivar, Dídac Mauricio

**Affiliations:** ^1^Primary Health Care Center Raval, Gerència d'Àmbit d'Atenció Primària Barcelona Ciutat, Institut Catala de la Salut, Avenida Drassanes 17-21, 08001 Barcelona, Spain; ^2^Unitat de Suport a la Recerca Barcelona Ciutat, Institut Universitari de Investigació en Atenció Primària Jordi Gol (IDIAP Jordi Gol), Avenida Gran Via de les Corts Catalanes, 587 Àtic, 08007 Barcelona, Spain; ^3^Primary Health Care Center La Mina, Gerència d'Àmbit d'Atenció Primària Barcelona Ciutat, Institut Catala de la Salut, C/Mar s/n, Sant Adrià de Besòs, 08930 Barcelona, Spain; ^4^Department of Endocrinology and Nutrition, Diabetes Unit, Hospital Clinic, University of Barcelona, C/Villarroel 170, 08036 Barcelona, Spain; ^5^Department of Endocrinology & Nutrition, Health Sciences Research Institute and Hospital Universitari Germans Trias i Pujol, Carretera Canyet s/n, 08916 Badalona, Spain

## Abstract

The objective of this cross-sectional study was to assess differences in the control and treatment of modifiable cardiovascular risk factors (CVRF: HbA1c, blood pressure [BP], LDL-cholesterol, body mass index, and smoking habit) according to gender and the presence of cardiovascular disease (CVD) in patients with type 2 diabetes mellitus (T2DM) in Catalonia, Spain. The study included available data from electronic medical records for a total of 286,791 patients. After controlling for sex, age, diabetes duration, and treatment received, both men and women with prior CVD had worse cardiometabolic control than patients without previous CVD; women with prior CVD had worse overall control of CVRFs than men except for smoking; and women without prior CVD were only better than men at controlling smoking and BP, with no significant differences in glycemic control. Finally, although the proportion of women treated with lipid-lowering medications was similar to (with prior CVD) or even higher (without CVD) than men, LDL-cholesterol levels were remarkably uncontrolled in both women with and women without CVD. The results stress the need to implement measures to better prevent and treat CVRF in the subgroup of diabetic women, specifically with more intensive statin treatment in those with CVD.

## 1. Introduction

The prevalence rates of diabetes mellitus (DM) have significantly increased during the last years, accompanied by a parallel rise in complications and deaths from the disease [1, 2]. The worldwide prevalence in 2013 has been estimated to be 8.3%, and it is expected to be about 1 adult in 10 by 2035, which represents a substantial 55% increase [3, 4]. A recent population-based survey conducted in Spain reported a global prevalence of DM of 13.8% in adult subjects, and 43.5% of them were unaware of their disease, thus corresponding to a prevalence of unknown DM of 6% [5].

People with type 2 diabetes mellitus (T2DM) are at increased risk of cardiovascular complications such as coronary artery disease, stroke, or peripheral vascular disease [6, 7]. In turn, these complications are associated with increased morbidity and mortality and have a detrimental effect on health-related quality of life [8]. Current available evidence indicates that, in terms of risk reduction of cardiovascular and microvascular complications, control of blood pressure and lipid levels is more effective than glucose control [9–11]. Additionally, type 2 diabetic patients with clinical cardiovascular disease (CVD) are at a higher risk of a recurrent cardiovascular event [12, 13]. However, several studies have shown that, in clinical practice, secondary prevention strategies in diabetic patients with CVD are associated with a suboptimal cardiometabolic control [11, 14].

Systematic reviews of the literature have reported that the excess relative risk of CVD attributable to diabetes is 2-fold in men and 3- to 4-fold in women [15, 16], and this has been further confirmed by several meta-analyses [17–19]. Some authors have postulated that diabetes prompts the loss of the natural hormonal protection against CVD in women [20, 21], but several factors that may explain this excess risk in women relative to men have been identified so far [22–25], and they mainly include a low risk perception by health care providers [26]; an increased time to proper medical care from the onset of symptoms; a lower predictive capability of certain diagnostic tests (e.g., stress test); a differential drug response among women to some medications such as aspirin [27] or statins [28], which decreases their effectiveness; and worse clinical outcomes after therapeutic procedures [29].

Cross-sectional studies have reported that the control of cardiovascular risk factors (CVRF) is poorer among diabetic women relative to diabetic men of the same age [30–32]. Moreover, the follow-up of the population in the National Health and Nutrition Examination Surveys (NHANES) has shown that, for the past years, there has been an overall decline in mortality rates due to CVD, but not in the subgroup of diabetic women [33].

On the other hand, studies derived from the analysis of large databases have proven to be useful for evaluating cardiometabolic control, associated risk factors, long-term complications, and other clinically relevant aspects of T2DM [34–37].

The aim of the present population-based study was to assess differences in the degree of control and treatment of modifiable CVRF according to gender and CVD in patients with T2DM in Catalonia, Spain.

## 2. Materials and Methods

### 2.1. Design

This cross-sectional study includes all type 2 diabetes subjects visiting any of the 274 primary care centres pertaining to the Catalan Health Institute (ICS) in Catalonia, a northeastern region of Spain, which takes care of a population of about 5.8 million patients (80% of the total population for the region).

The data for the present study (eCONTROL) were extracted from SIDIAP (Information System for the Development of Research in Primary Care; SIDIAP) [38], a database of electronic medical records started in 2006. Methodological details of the study of diabetes mellitus using this database have been described in previous publications [36, 39]. Briefly, SIDIAP contains anonymized longitudinal patient information obtained through use of specific software (eCAP) implemented in all primary care centers in Catalonia and includes sociodemographic characteristics, morbidity (by means of International Classification of Diseases codes; ICD-10), clinical and lifestyle variables, specialist referrals, and results of laboratory tests and treatments based on prescription- and pharmacy-invoicing data provided by the CatSalut general database.

### 2.2. Data Extraction

Data from patients attended before July 1, 2009, aging 31 to 90 years, and with a diagnosis of type 2 diabetes (ICD-10 codes E11 or E14) were extracted from the SIDIAP database [36]. Available variables (registered up to the end of 2009) included age; gender; time since diagnosis; estimated glomerular filtration rate (eGFR) using the Modified Diet in Renal Disease (MDRD) formula; standardized glycated haemoglobin (HbA1c) values, using the most recent value of the preceding 15 months; presence of cardiovascular disease, including coronary artery disease (ICD-10 codes I20, I21, I22, I23, or I24), stroke (ICD-10 codes I63, I64, G45, or G46), and peripheral artery disease (ICD-10 code I73.9); risk factors, including body mass index (BMI) (most recent value in the last 24 months), cholesterol levels (total, low-density lipoproteins or LDL-cholesterol, and high-density lipoproteins or HDL-cholesterol; most recent value in the last 15 months), blood pressure (BP) (systolic and diastolic mean value in the last 12 months), smoking status (most recent value); and data on prescribed glucose-lowering, lipid-lowering, and antihypertensive and antithrombotic medications.

Diagnostic criteria for CVRF were HbA1c > 7%; hypertension (blood pressure > 140/90 mmHg); hypercholesterolemia (total cholesterol > 250 mg/dL); hypertriglyceridemia (triglycerides > 150 mg/dL); obesity (BMI > 30 kg/m^2^); and current or former smoking habit. Treatment goals for patients with and without a history of CVD were based on local guidelines [40, 41]; without CVD prevention: HbA1c ≤ 7%, BP ≤ 140/90 mmHg, and LDL-cholesterol ≤ 130 mg/dL; with CVD prevention: HbA1c ≤ 7%, BP ≤ 140/90 mmHg, and LDL-cholesterol ≤ 100 mg/dL.

This study was approved by the Ethics Committee of the Primary Health Care University Research Institute (IDIAP) Jordi Gol.

### 2.3. Statistical Analysis

Descriptive analyses were summarized by mean and standard deviation for continuous variables and percentages for categorical variables. Comparisons by gender and presence of CVD were performed with Pearson chi-square tests for categorical variables and analysis of variance (ANOVA) for continuous variables. We applied multilevel logistic regression models to identify the factors associated with good cardiometabolic control of CVRFs. Only those variables with a statistically significant effect (*P* < 0.05) in the univariate analyses were retained for the multivariate model. Analyses were performed stratifying according to the presence of CVD, and odds ratios (OR) and 95% confidence intervals (95% CI) were adjusted for gender, diabetes duration, and treatment as confounding variables. Statistical calculations were performed using StataCorp 2009 (Stata Statistical Software: Release 11. College Station, TX: StataCorp, LP).

## 3. Results

The study included data from a total of 286,791 patients with T2DM (153,987 men and 132,804 women). Overall, 18.4% of the patients (*N* = 52, 665) had a previous history of any CVD, which was more frequently reported among men (22.3% versus 13.8%).

In the overall population, all studied variables showed significant differences between men and women; women were in average older than men, had a longer duration of the disease, and had slightly worse cardiometabolic control than men, with higher blood pressure levels (mean 137.5/76.2 mmHg versus 136.9/76.6 mmHg; *P* < 0.005), higher LDL-cholesterol levels (mean 115.6 mg/dL versus 109.7 mg/dL; *P* < 0.005), and higher average BMI (mean 30.5 versus 28.8; *P* < 0.005), but slightly better control of the percentage of HbA1c than men (7.1% versus 7.2%; *P* < 0.005) (Table 1). Moreover, triglyceride levels were lower in women (mean 153.5 mg/dL versus 158.5 mg/dL; *P* < 0.005), and there were far more nonsmokers among diabetic women (88% versus 43.5%; *P* < 0.005).

### 3.1. Cardiometabolic Control of T2DM and Degree of Control of CVRF according to History of CVD and Gender

The stratified analysis according to history of CVD showed that men with prior CVD had significantly better control of BP, weight, lipid profile, and smoking than men without a history of CVD (all variables *P* < 0.001) (Table 1). Additionally, there were no clinically significant differences with regard to glycemic control between the groups (*P* = 0.058). However, this pattern was strikingly different among women: those with previous CVD had significantly higher HbA1c (7.2% versus 7.1%; *P* = 0.003), systolic BP (mean 138 mmHg versus 137.5 mmHg; *P* < 0.001), and triglyceride values (156.3 mg/dL versus 153.1 mg/dL; *P* < 0.001) than women without a history of CVD.

**Figure 1:**
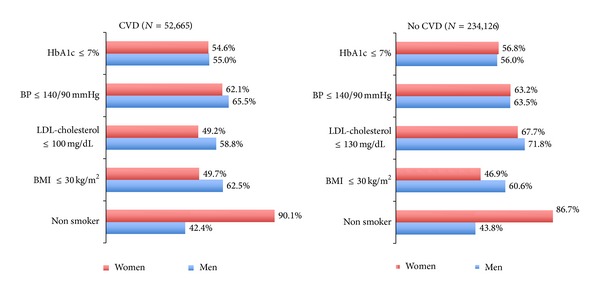
Percentage of patients with T2DM and good control of CVRF by gender and history of CVD (all variables showed significant differences between sexes (*P* < 0.005) and between CVD and no CVD in both men and women (*P* < 0.001), except for HbA1c: *P* = 0.058 in men and *P* = 0.003 in women. BMI, body mass index; BP, blood pressure; CVD, cardiovascular disease).

When considering the adequate treatment goals of CVRFs by gender, women showed worse overall control than men (*P* < 0.005 for all variables except for smoking); this was seen both in subjects with no previous CVD and in those with history of any CVD (*P* < 0.001 for all studied variables) (Table 1 and Figure 1). The greatest differences compared with men were seen in the levels of LDL-cholesterol and in weight, while differences in BP were less evident, and the percentage of patients with HbA1c ≤ 7% was slightly higher among women without CVD (56.8% versus 56%, *P* < 0.05) and lower in women with CVD (54.6% versus 55%, *P* < 0.05). In accordance, the degree of composite control of CVRFs, that is, simultaneously taking into account hyperglycemia (HbA1C ≤ 7%, BP ≤ 140/90 mmHg) and LDL-cholesterol levels (LDL-cholesterol ≤ 130 mg/dL in patients without previous CVD and ≤100 mg/dL in those with prior CVD), was significantly worse among women: 25.1% of women without CVD were in good control compared to 27% of men, and among those with prior CVD, 17.7% of women had an optimal composite control versus 22.8% of men (*P* < 0.005 in both cases). Moreover, the proportion of patients with good composite control of CVRFs was lower among those with prior CVD, and this was true for both men and women: 17.7% of women with prior CVD were in good control versus 21.5% without CVD (*P* < 0.001), and 22.8% of men with prior CVD were in good control versus 27% without CV (*P* < 0.001).

### 3.2. Multivariate Analysis of Good CVRF Control according to Gender and CVD

After adjusting for gender (woman), age, diabetes duration, and treatment received, multivariate analysis showed that men in secondary prevention after CVD had better control of all risk parameters except for smoking. In the case of prevention of CVD, women still had better control over smoking than men, but also better control of their BP, whilst there were no clinically significant differences in glycemic control between genders (Table 2), and women remained worse than men at controlling weight and LDL-cholesterol levels.

### 3.3. Degree of CVRF Control in Different CVDs

Study of the different macrovascular complications, specifically coronary heart disease (CHD), stroke, or peripheral arterial disease (PAD), showed that the proportion of women with good control of target CVRFs, namely, HbA1c ≤ 7%, BP ≤ 140/90 mmHg, and BMI ≤ 30 Kg/m^2^, and also lipid profiles in subjects with or without prior CVD was lower than men irrespective of the type of CVD (*P* < 0.001 in all cases) (Table 3).

### 3.4. Treatment of CVRFs in Patients with and without CVD according to Gender

We further studied whether treatment for the different CVRFs differed between genders in the presence/absence of prior CVD (Table 4). Among the subset of patients without a history of CVD, women had higher rates of prescribed glucose-lowering, antihypertensive, and lipid-lowering drugs than men (75% versus 73.3%, 70.8% versus 59.9%, and 47.6% versus 43.1%, resp.) and similar use of antiplatelet agents (27.6% versus 28.3%). However, in the subgroup of patients with a history of CVD, differences in the use or intensity of glucose-lowering and lipid-lowering treatments were not clinically relevant between genders, but women used less antiplatelet agents (71.8% versus 77.5%) and more antihypertensive agents (88.4% versus 86.4%) than men. Of note was that oral glucose-lowering agents in mono- or combined therapy were less often prescribed to women than to men in favor of a greater use of insulin therapy, either alone or combined with oral glucose-lowering drugs.

## 4. Discussion

Gender differences among the diabetic population include disparities in adherence to treatment [42], in control of cardiometabolic parameters and risk of CVD [30, 31, 43], and also in the therapeutic management of cardiovascular risk factors [25, 44, 45].

The prevalence rates of T2DM and CVD in our study were higher among men, which is in line with previous population-based studies [30, 46–48], although rates vary depending on the age range, country, and definition of CVD.

The results of the study showed that there were significant gender differences in the control of T2DM and CVD individual risk factors. Namely, compared with men, women were on average older and had a longer duration of disease, and apart from less frequently being smokers than men, they had poorer control of hypertension, LDL-cholesterol levels, and BMI. This profile of worse control of CVRFs has been consistently reported before in previous surveys conducted in Spain and in other countries [30–32, 39, 43, 49–52], but the present study is the largest one ever performed in real-life clinical practice. Moreover, the proportion of women who achieved the target of stipulated recommendations to control the risk of CVD in our study was lower than men except for glycemic control, and the composite control of multiple risk outcomes (Hb1Ac, BP, and LDL-cholesterol simultaneously) was also poorer among women. These results are also in agreement with the above mentioned studies and with results from studies specifically assessing gender differences in composite risk factors in T2DM [43, 53], which have found that women are approximately 3 times less likely to achieve combined cardiometabolic control than men [43].

There are few reports assessing the control of CVRFs in T2DM according to gender as well as for the presence of prior CVD, and the present study is the first one conducted in a Spanish population. Our analysis stratifying by presence of prior CVD showed that both men and women with CVD in general had poorer control of CVRFs than those without. As for the degree of control of modifiable CVRFs, multivariate analysis showed that women with prior CVD were less likely to achieve their therapeutic targets than men for all parameters except for smoking. Women without CVD achieved the recommended HbA1c target as optimally as men and were better at controlling BP and smoking but again more frequently did not achieve recommended therapeutic targets for obesity and LDL-cholesterol. Our results on patients with prior CVD are in agreement with a previous cross-sectional study conducted in Germany, which found that women were more likely to have uncontrolled systolic BP, LDL-cholesterol, and HbA1c levels [25]; similarly, another cross-sectional analysis conducted in the US found that women were more liable to have suboptimal control of systolic BP and LDL-cholesterol but found no differences in glycemic control relative to men [45]. As for patients without prior CVD, the US study found no significant differences in the degree of control of any studied modifiable CVRF [45], and the German study only found a higher probability of women having uncontrolled LDL-cholesterol relative to men [25], which is in agreement with our results, although we also found that women had even better control of BP than men. Unfortunately, our results on smoking and BMI cannot be compared with these 2 studies, since both of them included these 2 risk factors as confounding covariates in their analysis.

There is compelling evidence in Spain and other countries that women receive less health care attention not only for the treatment of their T2DM [54], but also for the prevention and treatment of associated CV complications [14, 19, 25, 26, 30, 44, 45], as women both with and without CVD receive less lipid-lowering and antithrombotic therapy than men [29, 47, 55, 56]. Studies stratifying by gender and comorbid CVD are scarce but concur that women are less intensively treated with lipid-lowering drugs than men, in patients both with and without prior CVD, while findings on gender disparities according to prior CVD regarding the use or intensity of treatment with antihypertensive or glucose-lowering drugs are inconsistent across reports [25, 45, 47]. Differences between studies may be due to genetic or ethnic differences, geographical variations in access to available health care resources, different ambulatory physician practices between countries, and disparities in the economic barriers to care due to the type of insurance (public or private) paying for the treatment.

When we assessed whether there were gender disparities in the management of modifiable CVRF in T2DM patients according to a history of CVD, we found that women were more likely to be treated with antihypertensive drugs and less likely to take antiplatelet drugs than men irrespective of having a history of CVD, while glucose- and lipid-lowering treatment varied according to the absence/presence of prior CVD: the proportion of women with CVD taking glucose and/or lipid-lowering medications was similar to men, but women without CVD took more glucose and/or lipid-lowering drugs than men. However, while the degree of achieved glycemic control was similar between women with and without previous CVD, lipid levels were remarkably uncontrolled in both cases and more pronounced in women with prior CVD. This is of concern if we take into account that a history of CVD is an independent factor associated with higher morbidity and mortality and that the 4-year survival rate of women with prior CVD is lower than in women without a history of CVD [30]. Moreover, the fact that women without prior CVD did not achieve adequate control of lipid levels, in spite of being more likely to be treated with lipid-lowering medications than men, could be related to the use of less intensive therapy or to a differential response to statins relative to men, although this is controversial in the case of primary prevention [57, 58]. With regard to the degree of control of BP, we observed that women without CVD had similar control to men, in spite of higher levels of treatment with antihypertensive drugs, while women with CVD still had uncontrolled BP relative to men although they were treated in a comparable proportion, an observation that has been previously reported [29]. This is also of concern if we consider that women have a higher lifetime risk of stroke than men, in part because they have a longer life expectancy and because the risk of stroke increases with age [59], therefore, underlining the need for more intensive or proper control of BP. Taken together, our results show that the treatment and control of the 2 parameters that most effectively prevent CVD, namely, BP and lipid levels, remain a challenge (particularly LDL-cholesterol levels) in the case of women with T2DM and a history of CVD.

Strengths of the present study include the use of registries coming from primary care medical records, which allows the collection of a large volume of patients' real-life clinical practice data. However, there are some limitations that should be acknowledged and considered when interpreting the results of this study. Firstly, inherent to any cross-sectional design, no causal associations or conclusion on trends in treatment can be drawn, and the retrospective design is subject to biases concerning the lack of data recording for some of the studied variables (e.g., 25% of patients did not have corresponding HbA1c values for the previous year). Secondly, the studied cohort is representative of a specific territory in Spain and may not necessarily reflect standards of care in other territories. Thirdly, information on treated (and the specific therapeutic agents prescribed) and untreated patients was based on drugs obtained at the pharmacy, and we were not able to assess medication adherence factors. Finally, we had no data to assess factors known to differ by gender in T2DM that may influence disease outcomes, such as diabetes knowledge, self-management practices, lifestyle related factors, socioeconomic status, education, or social support [51].

## 5. Conclusions

The results of the study confirm that Spanish women with T2DM have suboptimal control of CVRFs; they also show that compared with men women with CVD were less likely to achieve therapeutic goals for BMI, BP, LDH-cholesterol, and HbA1c and that those without a history of CVD were also less likely to achieve BMI and LDL-cholesterol recommended goals. Furthermore, although the proportion of women treated with lipid-lowering medications was similar to or even higher than men, LDL-cholesterol levels were remarkably uncontrolled in both women with and without CVD, and women with CVD still had uncontrolled BP relative to men in spite of being treated with antihypertensive drugs in a comparable proportion of cases. The observed differences have clinical implications that warrant further investigation through studies specifically designed to assess gender differences in the control of modifiable CVRF and further stress the need to implement measures to better prevent and treat this subgroup of diabetic women. Actions should include not only targeted awareness programs for health professionals, but also the implementation of specific educational programs aimed at improving self-awareness and self-care in women with T2DM.

## Figures and Tables

**Table 1:** Demographic, clinical characteristics, and degree of cardiometabolic control by gender and presence of CVD*.

	All patients		CVD		No CVD	
	Men	Women	Men	Women	Men	Women
	*N* = 153,987	*N* = 132,804	*N* = 34,283	*N* = 18,382	*N* = 119,704	*N* = 114,422
CV risk factors						
Age, mean (SD), years	66.4 (11.3)	70.3 (11.1)	70.9 (9.6)	75.6 (8.7)	65.1 (11.4)	69.4 (11.2)
Diabetes duration, mean (SD), years	6.2 (4.8)	6.9 (5.3)	7.3 (5.5)	8.3 (6.4)	5.9 (4.5)	6.7 (5.1)
Hypertension, %	58.6	69.7	69.5	81.5	55.4	67.8
Systolic BP, mean (SD), mmHg	136.9 (13.6)	137.5 (14)	136.1 (14.3)	138 (14.7)	137.2 (13.4)	137.5 (13.8)
Diastolic BP, mean (SD), mmHg	76.6 (8.5)	76.2 (8.1)	73.8 (8.4)	73.6 (8.2)	77.5 (8.3)	76.6 (8)
Diabetic retinopathy, %	5.6	6.1	8.3	10.9	4.8	5.4
Diabetic nephropathy, %	20.7	12.3	26.7	18.3	19	11.3
BMI, mean (SD), kg/m^2^	28.8 (4.3)	30.5 (5.6)	28.6 (4.1)	30.1 (5.4)	28.9 (4.3)	30.6 (5.6)
HbA1c, %	7.2 (1.5)	7.1 (1.4)	7.1 (1.4)	7.2 (1.4)	7.2 (1.5)	7.1 (1.4)
Total cholesterol, mean (SD), mg/dL	186.2 (38.2)	198.4 (38)	171.5 (36.9)	185.6 (39.4)	190.5 (37.5)	200.4 (37.3)
HDL-cholesterol, mean (SD), mg/dL	46.2 (12.3)	52.7 (13.4)	44.4 (11.8)	50.2 (12.9)	46.7 (12.4)	53.1 (13.4)
LDL-cholesterol, mean (SD), mg/dL	109.7 (32.2)	115.6 (32.3)	97.1 (30.7)	104.4 (32.5)	113.6 (31.6)	117.4 (31.9)
Triglycerides, mean (SD), mg/dL	158.5 (117.3)	153.5 (88.7)	153.4 (106.8)	156.3 (91.8)	160 (120.3)	153.1 (88.2)
Smoking status, %						
Non smokers	43.5	88	42.4	90.1	43.8	87.6
Current smokers	23.9	6.2	18.1	3.8	25.6	6.6
Ex-smoker	32.6	5.8	39.5	6.1	30.6	5.8
Degree of CVRF control						
HbA1c ≤ 7%, %	55.8	56.5	55	54.6	56	56.8
BMI ≤ 30 kg/m^2^, %	61	47.3	62.5	49.7	60.6	46.9
BP ≤ 140/90 mmHg, %	63.9	63.1	65.5	62.1	63.5	63.2
LDL-cholesterol ≤ 130 mg/dL, % (PP)	75.2	69.4	86.3	80.2	71.8	67.7
LDL-cholesterol ≤ 100 mg/dL, % (SP)	41.3	34.2	58.8	49.2	35.9	31.7
HbA1c ≤ 7% + BP ≤ 140/90 mmHg + LDL ≤ 130 mg/dL, %	28.5	25.6	33.1	28.9	27.0	25.1
HbA1c ≤ 7% + BP ≤ 140/90 mmHg + LDL ≤ 100 mg/dL, %	15.7	12.4	22.8	17.7	13.5	11.5

*All variables showed significant differences between sexes (*P* < 0.005) and between CVD and no CVD in both men and women (*P* < 0.001), except for HbA1c: *P* = 0.058 in men and *P* = 0.003 in women. BMI: body mass index; BP: blood pressure; CVD: cardiovascular disease; PP: primary prevention; SD: standard deviation; SP: secondary prevention.

**Table 2:** Multivariate analysis on the degree of control of CVRFs stratified according to the presence of CVD.

	CVD		No CVD	
	OR^a^ (95% CI)∗	*P* value	OR^a^ (95% CI)∗	*P* value
HbA1c ≤ 7%	0.95 (0.91–1.00)	0.041	1.01 (0.99–1.03)	0.23
PA ≤ 140/90 mmHg	0.879 (0.84–0.92)	<0.001	1.082 (1.06–1.13)	<0.001
LDL-cholesterol				
≤130 mg/dL (CVD)	0.67 (0.64–0.70)	<0.001	0.74 (0.72–0.76)	<0.001
≤100 mg/dL (no CVD)				
BMI ≤ 30 Kg/m^2^	0.50 (0.48–0.52)	<0.001	0.53 (0.52–0.54)	<0.001
Nonsmoker	4.20 (3.86–4.58)	<0.001	4.01 (3.39–4.13)	<0.001

BMI: body mass index; BP: blood pressure; CVD: cardiovascular disease; OR: odds ratio.

∗OR^a^: odds ratio adjusted by age, diabetes duration, treatment received, and sex (women).

**Table 3:** Degree of CVRFs control (% and 95% CI) in different macrovascular complications according to sex.

	CHD		Stroke		PAD		CHD + stroke		CHD + PAD		Stroke + PAD		CHD + stroke + PAD	
	*N* = 32,313		*N* = 18,768		*N* = 8,420		*N* = 3,670		*N* = 2,312		*N* = 1,265		*N* = 411	
	Men	Women	Men	Women	Men	Women	Men	Women	Men	Women	Men	Women	Men	Women
HbA1c ≤ 7%	54.2 (53.4–54.9)	52.8 (51.7–53.9)	58.1 (57–59.1)	57.2 (56.0–58.5)	50.5 (49.1–51.9)	48.3 (45.8–50.8)	53.8 (51.5–56.0)	52.0 (48.8–55.2)	49.4 (46.8–52.0)	44.5 (39.0–50.1)	51.9 (48.5–55.4)	43.5 (36.5–50.6)	51.3 (45.3–57.4)	46.8 (32.0–61.6)
BP ≤ 140/90 mmHg	67.3 (66.0–66.7)	62.6 (61.7–63.6)	64.2 (63.3–65.2)	63.0 (61.9–64.2)	61.0 (59.8–62.3)	55.8 (53.5–58.1)	65.8 (63.8–67.8)	64.5 (61.1–67.4)	65.0 (62.8–67.3)	59.0 (53.9–64.0)	63.2 (60.0–66.3)	56.0 (49.5–62.5)	65.0 (59.7–70.4)	55.7 (42.9–68.6)
Nonsmoker	43.8 (43.1–44.4)	90.5 (89.9–91.0)	45.2 (44.2–46.2)	90.8 (90.1–91.4)	30.7 (29.6–31.9)	85.7 (84.1–87.3)	45.1 (43.1–47.2)	91.8 (90.2–93.4)	34.3 (32.1–36.5)	89.3 (86.2–92.5)	33.1 (30.1–36.1)	87.6 (83.3–91.8)	37.5 (32.2–42.9)	88.5 (80.3–96.8)
BMI ≤ 30 kg/m^2^	39.7 (38.9–40.5)	52.4 (51.2–53.5)	34.3 (33.3–35.4)	47.8 (46.4–49.2)	33.0 (31.7–34.4)	47.7 (45.0–50.3)	34.0 (31.8–36.2)	50.8 (47.2–54.3)	35.9 (33.4–38.4)	47.3 (41.3–53.2)	31.9 (28.6–35.3)	44.0 (36.2–51.8)	31.1 (25.4–36.8)	53.5 (38.0–69.0)
LDL ≤ 130 mg/dL	89.1 (88.6–89.5)	82.8 (82.0–83.7)	84.9 (84.1–85.7)	78.9 (77.8–79.9)	82.2 (81.1–83.2)	73.7 (71.4–75.9)	90.9 (89.6–92.9)	84.4 (82.0–86.8)	89.8 (88.2–91.4)	81.5 (76.9–86.0)	86.1 (83.6–88.5)	79.0 (73.0–85.0)	88.0 (84.0–92.1)	81.0 (68.6–93.3)
LDL ≤ 100 mg/dL	63.1 (62.3–63.8)	53.2 (52.0–54.3)	56.4 (55.3–57.5)	46.8 (45.5–48.1)	51.8 (50.4–53.2)	42.2 (39.6–44.7)	65.1 (62.9–67.3)	54.7 (51.4–57.9)	62.4 (59.8–65.0)	58.4 (52.6–64.1)	58.4 (54.9–62.0)	48.6 (41.3–56.0)	59.8 (53.7–65.9)	54.8 (39.1–70.5)

BMI: body mass index; BP: blood pressure; CHD: coronary heart disease; PAD: peripheral artery disease.

**Table 4:** Treatment (%) used to control the different CVRFs in patients with or without CVD by gender.

Treatment	All patients			CVD			No CVD		
	*N* = 286,791			*N* = 52,665			*N* = 234,126		
	Men	Women	*P* value	Men	Women	*P* value	Men	Women	*P* value
	*N* = 153,987	*N* = 132,804		*N* = 34,283	*N* = 18,382		*N* = 119,704	*N* = 114,422	
Glucose-lowering									
Lifestyle changes only	24.6	24.1	0.003	17.8	18.2	0.021	26.6	25.1	<0.001
Oral monotherapy	36.3	34.5	<0.001	33.8	29.2	<0.001	37.0	35.4	<0.001
Combination of OAD	22.9	21.9	<0.001	23.5	19.9	<0.001	22.7	22.2	0.001
Insulin + OAD	8.80	11.7	<0.001	13.3	18.1	<0.001	7.50	10.7	<0.001
Insulin monotherapy	7.37	7.80	<0.001	11.7	14.7	<0.001	6.10	6.70	<0.001
Any pharmacological treatment	*75.4 *	*75.9 *	*0.003 *	*82.3 *	*81.9 *	*0.021 *	*73.3 *	*75 *	*<0.001 *
Antihypertensive									
No treatment	34.2	26.8	<0.001	13.6	11.6	<0.001	40.1	29.2	<0.001
ACE inhibitor/ARA2	16.3	14.9	<0.001	12.3	11.4	0.004	17.5	15.5	<0.001
Diuretic	2.01	4.4	<0.001	1.37	2.45	<0.001	2.20	4.72	<0.001
Beta-blocker	2.59	1.91	<0.001	5.60	3.37	<0.001	1.73	1.68	0.04
Calcium-channel blocker	2.19	2.15	0.43	3.82	3.28	0.001	1.73	1.97	<0.001
Combination of 2	22.7	26.8	<0.001	30.4	29.8	0.17	20.5	26.3	<0.001
Combination of 3 or more	19.5	22.8	<0.001	32.6	37.9	<0.001	15.7	20.3	<0.001
Any pharmacological treatment	*65.8 *	*73.2 *	*<0.001 *	*86.4 *	*88.4 *	*<0.001 *	*59.9 *	*70.8 *	*<0.001 *
Lipid-lowering									
No treatment	50.3	49.4	<0.001	27.1	31.1	<0.001	56.9	52.4	<0.001
Statin	40.5	43.3	<0.001	60.2	58.9	0.003	34.8	40.8	<0.001
Fibrate	3.88	2.81	<0.001	2.36	2.17	0.17	4.31	2.91	<0.001
Statin + fibrate	1.06	0.67	<0.001	1.81	1.13	<0.001	0.84	0.59	<0.001
Any pharmacological treatment	*49.7 *	*50.6 *	*<0.001 *	*72.9 *	*68.9 *	*<0.001 *	*43.1 *	*47.6 *	*<0.001 *
Antiplatelet									
No treatment	60.7	66.3	<0.001	22.5	28.2	<0.001	71.7	72.4	<0.001
Aspirin	31.4	28.8	<0.001	52.2	51.2	0.02	25.4	25.2	0.19
Clopidogrel	4.14	2.98	<0.001	12.6	11.9	0.01	1.71	1.55	0.004
Any pharmacological treatment	*39.3 *	*33.7 *	*<0.001 *	*77.5 *	*71.8 *	*<0.001 *	*28.3 *	*27.6 *	*<0.001 *

ACE: acetylcholinesterase; ARA2: angiotensin II receptor antagonist; BMI: body mass index; BP: blood pressure; CVD: cardiovascular disease; OAD: oral antidiabetic drugs.

## References

[1] Shaw JE, Sicree RA, Zimmet PZ (2010). Global estimates of the prevalence of diabetes for 2010 and 2030. *Diabetes Research and Clinical Practice*.

[2] Whiting DR, Guariguata L, Weil C, Shaw J (2011). IDF Diabetes Atlas: global estimates of the prevalence of diabetes for 2011 and 2030. *Diabetes Research and Clinical Practice*.

[3] Guariguata L, Whiting DR, Hambleton I (2014). Global estimates of diabetes prevalence for 2013 and projections for 2035. *Diabetes Research and Clinical Practice*.

[4] International Diabetes Federation (2013). *IDF Diabetes Atlas*.

[5] Soriguer F, Goday A, Bosch-Comas A (2012). Prevalence of diabetes mellitus and impaired glucose regulation in Spain: the Di@bet.es Study. *Diabetologia*.

[6] Donahue RP, Orchard TJ (1992). Diabetes mellitus and macrovascular complications: an epidemiological perspective. *Diabetes Care*.

[7] Booth GL, Kapral MK, Fung K, Tu JV (2006). Relation between age and cardiovascular disease in men and women with diabetes compared with non-diabetic people: a population-based retrospective cohort study. *The Lancet*.

[8] Leal J, Gray AM, Clarke PM (2009). Development of life-expectancy tables for people with type 2 diabetes. *European Heart Journal*.

[9] Gæde P, Vedel P, Larsen N, Jensen GVH, Parving H-H, Pedersen O (2003). Multifactorial intervention and cardiovascular disease in patients with type 2 diabetes. *The New England Journal of Medicine*.

[10] Holman RR, Paul SK, Bethel MA, Matthews DR, Neil HAW (2008). 10-Year follow-up of intensive glucose control in type 2 diabetes. *The New England Journal of Medicine*.

[11] Mannucci E, Dicembrini I, Lauria A, Pozzilli P (2013). Is glucose control important for prevention of cardiovascular disease in diabetes?. *Diabetes Care*.

[12] American Diabetes Association (2014). Standards of medical care in diabetes—2014. *Diabetes Care*.

[13] Giorda CB, Avogaro A, Maggini M (2008). Recurrence of cardiovascular events in patients with type 2 diabetes: epidemiology and risk factors. *Diabetes Care*.

[14] Jørgensen CH, Gislason GH, Ahlehoff O, Andersson C, Torp-Pedersen C, Hansen PR (2014). Use of secondary prevention pharmacotherapy after first myocardial infarction in patients with diabetes mellitus. *BMC Cardiovascular Disorders*.

[15] Orchard TJ (1996). The impact of gender and general risk factors on the occurrence of atherosclerotic vascular disease in non-insulin-dependent diabetes mellitus. *Annals of Medicine*.

[16] Kanaya AM, Grady D, Barrett-Connor E (2002). Explaining the sex difference in coronary heart disease mortality among patients with type 2 diabetes mellitus: a meta-analysis. *Archives of Internal Medicine*.

[17] Lee WL, Cheung AM, Cape D, Zinman B (2000). Impact of diabetes on coronary artery disease in women and men: a meta- analysis of prospective studies. *Diabetes Care*.

[18] Hu FB, Stampfer MJ, Solomon CG (2001). The impact of diabetes mellitus on mortality from all causes and coronary heart disease in women: 20 years of follow-up. *Archives of Internal Medicine*.

[19] Huxley R, Barzi F, Woodward M (2006). Excess risk of fatal coronary heart disease associated with diabetes in men and women: meta-analysis of 37 prospective cohort studies. *British Medical Journal*.

[20] Castelli WP (1988). Cardiovascular disease in women. *American Journal of Obstetrics and Gynecology*.

[21] Dantas APV, Fortes ZB, de Carvalho MHC (2012). Vascular disease in diabetic women: why do they miss the female protection?. *Experimental Diabetes Research*.

[22] Barrett-Connor EL, Cohn BA, Wingard DL, Edelstein SL (1991). Why is diabetes mellitus a stronger risk factor for fatal ischemic heart disease in women than in men? The Rancho Bernardo Study. *Journal of the American Medical Association*.

[23] Juutilainen A, Kortelainen S, Lehto S, Rönnemaa T, Pyörälä K, Laakso M (2004). Gender difference in the impact of type 2 diabetes on coronary heart disease risk. *Diabetes Care*.

[24] Marrugat J, Sala J, Aboal J (2006). Epidemiology of cardiovascular disease in women. *Revista Espanola de Cardiologia*.

[25] Gouni-Berthold I, Berthold HK, Mantzoros CS, Böhm M, Krone W (2008). Sex disparities in the treatment and control of cardiovascular risk factors in type 2 diabetes. *Diabetes Care*.

[26] Gutiérrez PC, Bejarano JML, Juanatey JRG, Núñez AG, Fernández FJP, Sardá AN (2003). Different approach in high-cardiovascular-risk women, compared to men: a multidisciplinary study-Spain. *Medicina Clinica*.

[27] Persell SD, Baker DW (2004). Aspirin use among adults with diabetes: recent trends and emerging sex disparities. *Archives of Internal Medicine*.

[28] Tonstad S, Rosvold EO, Furu K, Skurtveit S (2004). Undertreatment and overtreatment with statins: the Oslo Health Study 2000-2001. *Journal of Internal Medicine*.

[29] Wexler DJ, Grant RW, Meigs JB, Nathan DM, Cagliero E (2005). Sex disparities in treatment of cardiac risk factors in patients with type 2 diabetes. *Diabetes Care*.

[30] Vidal-Pérez R, Otero-Raviña F, Grigorian-Shamagian L (2010). Sex does not influence prognosis in diabetic patients. The Barbanza Diabetes study. *Revista Espanola de Cardiologia*.

[31] Franzini L, Ardigò D, Cavalot F (2013). Women show worse control of type 2 diabetes and cardiovascular disease risk factors than men: results from the MIND.IT Study Group of the Italian Society of Diabetology. *Nutrition, Metabolism and Cardiovascular Diseases*.

[32] Penno G, Solini A, Bonora E (2013). Gender differences in cardiovascular disease risk factors, treatments and complications in patients with type 2 diabetes: the RIACE Italian multicentre study. *Journal of Internal Medicine*.

[33] Gregg EW, Gu Q, Cheng YJ, Narayan KMV, Cowie CC (2007). Mortality trends in men and women with diabetes, 1971 to 2000. *Annals of Internal Medicine*.

[34] Morris AD, Boyle DIR, MacAlpine R (1997). The diabetes audit and research in Tayside Scotland (DARTS) study: electronic record linkage to create a diabetes register. *British Medical Journal*.

[35] Eliasson B, Cederholm J, Nilsson P, Gudbjörnsdóttir S (2005). The gap between guidelines and reality: type 2 diabetes in a national diabetes register 1996–2003. *Diabetic Medicine*.

[36] Vinagre I, Mata-Cases M, Hermosilla E (2012). Control of glycemia and cardiovascular risk factors in patients with type 2 diabetes in primary care in Catalonia (Spain). *Diabetes Care*.

[37] Sperl-Hillen JAM, O'Connor PJ (2005). Factors driving diabetes care improvement in a large medical group: ten years of progress. *American Journal of Managed Care*.

[38] Bolíbar B, Fina Avilés F, Morros R (2012). SIDIAP database: electronic clinical records in primary care as a source of information for epidemiologic research. *Medicina Clinica*.

[39] Mata-Cases M, Roura-Olmeda P, Berengué-Iglesias M (2012). Fifteen years of continuous improvement of quality care of type 2 diabetes mellitus in primary care in Catalonia, Spain. *International Journal of Clinical Practice*.

[40] Mata-Cases M, Cos-Claramunt FX, Morros R (2010). *Abordatge de la Diabetis Mellitus Tipus 2*.

[41] Ministerio de Sanidad y Consumo Guía de práctica clínica sobre diabetes tipo 2. http://www.guiasalud.es/viewGPC.asp?idGuia=429.

[42] Raum E, Krämer HU, Rüter G (2012). Medication non-adherence and poor glycaemic control in patients with type 2 diabetes mellitus. *Diabetes Research and Clinical Practice*.

[43] Strom Williams JL, Lynch CP, Winchester R (2014). Gender differences in composite control of cardiovascular risk factors among patients with type 2 diabetes. *Diabetes Technology and Therapeutics*.

[44] Krämer HU, Raum E, Rüter G (2012). Gender disparities in diabetes and coronary heart disease medication among patients with type 2 diabetes: results from the DIANA study. *Cardiovascular Diabetology*.

[45] Ferrara A, Mangione CM, Kim C (2008). Sex disparities in control and treatment of modifiable cardiovascular disease risk factors among patients with diabetes: Translating Research Into Action for Diabetes (TRIAD) study. *Diabetes Care*.

[46] Becker A, Bos G, de Vegt F (2003). Cardiovascular events in type 2 diabetes: comparison with nondiabetic individuals without and with prior cardiovascular disease: 10-Year follow-up of the Hoorn study. *European Heart Journal*.

[47] Baviera M, Santalucia P, Cortesi L (2014). Sex differences in cardiovascular outcomes, pharmacological treatments and indicators of care in patients with newly diagnosed diabetes: analyses on administrative database. *European Journal of Internal Medicine*.

[48] Mo F, Pogany LM, Li FCK, Morrison H (2006). Prevalence of diabetes and cardiovascular comorbidity in the Canadian Community Health Survey 2002–2003. *TheScientificWorldJournal*.

[49] Comaschi M, Coscelli C, Cucinotta D, Malini P, Manzato E, Nicolucci A (2005). Cardiovascular risk factors and metabolic control in type 2 diabetic subjects attending outpatient clinics in Italy: the SFIDA (survey of risk factors in Italian diabetic subjects by AMD) study. *Nutrition, Metabolism and Cardiovascular Diseases*.

[50] Winston GJ, Barr RG, Carrasquillo O, Bertoni AG, Shea S (2009). Sex and racial/ethnic differences in cardiovascular disease risk factor treatment and control among individuals with diabetes in the Multi-Ethnic Study of Atherosclerosis (MESA). *Diabetes Care*.

[51] Sandín M, Espelt A, Escolar-Pujolar A, Arriola L, Larrañaga I (2011). Desigualdades de género y diabetes mellitus tipo 2: la importancia de la diferencia. *Avances en Diabetología*.

[52] Sekerija M, Poljicanin T, Erjavec K, Liberati-Cizmek A-M, Prašek M, Metelko Z (2012). Gender differences in the control of cardiovascular risk factors in patients with type 2 diabetes -a cross-sectional study. *Internal Medicine*.

[53] Homko CJ, Zamora L, Santamore WP, Kashem A, McConnell T, Bove AA (2010). Gender differences in cardiovascular risk factors and risk perception among individuals with diabetes. *Diabetes Educator*.

[54] Correa-de-Araujo R, McDermott K, Moy E (2006). Gender differences across racial and ethnic groups in the quality of care for diabetes. *Women's Health Issues*.

[55] Ferrara A, Williamson DF, Karter AJ, Thompson TJ, Kim C (2004). Sex differences in qualify of health care related to ischemic heart disease prevention in patients with diabetes: the Translating Research Into Action for Diabetes (TRIAD) study, 2000-2001. *Diabetes Care*.

[56] Nau DP, Mallya U (2005). Sex disparity in the management of dyslipidemia among patients with type 2 diabetes mellitus in a managed care organization. *The American Journal of Managed Care*.

[57] Walsh JM, Pignone M (2004). Drug Treatment of Hyperlipidemia in Women. *Journal of the American Medical Association*.

[58] Mora S, Glynn RJ, Hsia J, MacFadyen JG, Genest J, Ridker PM (2010). Statins for the primary prevention of cardiovascular events in women with elevated high-sensitivity C-reactive protein or dyslipidemia: results from the justification for the use of statins in prevention: an intervention trial evaluating rosuvastatin (JUPITER) and meta-analysis of women from primary prevention trials. *Circulation*.

[59] Seshadri S, Beiser A, Kelly-Hayes M (2006). The lifetime risk of stroke: estimates from the framingham study. *Stroke*.

